# Larval ecology of mosquitoes in sylvatic arbovirus foci in southeastern Senegal

**DOI:** 10.1186/1756-3305-5-286

**Published:** 2012-12-07

**Authors:** Diawo Diallo, Cheikh T Diagne, Kathryn A Hanley, Amadou A Sall, Michaela Buenemann, Yamar Ba, Ibrahima Dia, Scott C Weaver, Mawlouth Diallo

**Affiliations:** 1 Unité d’entomologie médicale, Institut Pasteur de Dakar, Dakar, Sénégal; 2 Department of Biology, New Mexico State University, Las Cruces, New Mexico, United States of America; 3 Unité des arbovirus et virus des fièvres hémorragiques, Institut Pasteur de Dakar, Dakar, Sénégal; 4 Department of Geography, New Mexico State University, Las Cruces, New Mexico, United States of America; 5 Institute for Human Infections and Immunity, Center for Biodefense and Emerging Infectious Diseases, and Department of Pathology, University of Texas Medical Branch, Galveston, Texas, United States of America

**Keywords:** Mosquito larvae, Sylvatic arbovirus vectors, Microhabitats, Land covers, Species association,* Aedes furcifer*,*Aedes taylori*, *Aedes aegypti formosus*, Southeastern Senegal

## Abstract

**Background:**

Although adult mosquito vectors of sylvatic arbovirus [yellow fever (YFV), dengue-2 (DENV-2) and chikungunya (CHIKV)] have been studied for the past 40 years in southeastern Senegal, data are still lacking on the ecology of larval mosquitoes in this area. In this study, we investigated the larval habitats of mosquitoes and characterized their seasonal and spatial dynamics in arbovirus foci.

**Methods:**

We searched for wet microhabitats, classified in 9 categories, in five land cover classes (agriculture, forest, savannah, barren and village) from June, 2010 to January, 2011. Mosquito immatures were sampled monthly in up to 30 microhabitats of each category per land cover and bred until adult stage for determination.

**Results:**

No wet microhabitats were found in the agricultural sites; in the remaining land covers immature stages of 35 mosquito species in 7 genera were sampled from 9 microhabitats (tree holes, fresh fruit husks, decaying fruit husks, puddles, bamboo holes, discarded containers, tires, rock holes and storage containers). The most abundant species was *Aedes aegypti formosus,* representing 30.2% of the collections, followed by 12 species, representing each more than 1% of the total, among them the arbovirus vectors *Ae. vittatus* (7.9%), *Ae. luteocephalus* (5.7%), *Ae. taylori* (5.0%), and *Ae. furcifer* (1.3%). *Aedes aegypti, Cx. nebulosus, Cx. perfuscus, Cx. tritaeniorhynchus, Er. chrysogster* and *Ae. vittatus* were the only common species collected from all land covers. *Aedes furcifer* and *Ae. taylori* were collected in fresh fruit husks and tree holes. Species richness and dominance varied significantly in land covers and microhabitats. Positive associations were found mainly between *Ae. furcifer*, *Ae. taylori* and *Ae. luteocephalus.* A high proportion of potential enzootic vectors that are not anthropophilic were found in the larval mosquito fauna.

**Conclusions:**

In southeastern Senegal, *Ae. furcifer* and *Ae. taylori* larvae showed a more limited distribution among both land cover and microhabitat types than the other common species. Uniquely among vector species, *Ae. aegypti formosus* larvae occurred at the highest frequency in villages. Finally, a high proportion of the potential non-anthropophilic vectors were represented in the larval mosquito fauna, suggesting the existence of unidentified sylvatic arbovirus cycles in southeastern Senegal.

## Background

Southeastern Senegal (West Africa) is endemic for several arboviruses including dengue-2 and yellow fever (genus *Flavivirus*, family *Flaviviridae*) and chikungunya (genus *Alphavirus*, family *Togaviridae*) viruses that occur in sylvatic, enzootic transmission cycles between primates and arboreal mosquitoes [1-3]. The first evidence of sylvatic transmission of dengue-2 virus (DENV-2) in the area was virus isolation from a human in 1970 about 60 km from the Senegalese capital Dakar, and from pools of *Aedes luteocephalus* caught in a forest gallery near the town of Kédougou in southeastern Senegal [4]. After that, 5 amplifications of the sylvatic cycle were detected between 1980 and 2000 in Kédougou. During these amplifications, large numbers of DENV-2 strains were isolated from mosquitoes, mainly from *Ae. furcifer, Ae. luteocephalus*, *Ae. taylori*, *Ae. aegypti formosus*, and *Ae. vitattus*, one strain from the serum of a wild patas monkey (*Erythrocebus patas*), and four strains from human sera [5-8]. The sylvatic cycle of chikungunya virus (CHIKV) in southeastern Senegal is very similar to that of DENV-2. Indeed, although CHIKV has been isolated from 11 mosquito species and 3 different monkeys species during amplifications of the sylvatic cycle in the region, the data indicate that the main vectors (*Ae. furcifer, Ae. taylori, Ae. luteocephalus*) as well as vertebrate hosts (monkeys and humans) are the same as those for DENV-2. However, transmission of CHIKV may differ in subtle ways from that of sylvatic DENV-2, due to the possible existence of additional CHIKV vectors and vertebrate hosts other than monkeys, such as galagos (*Galago senegalensis*), palm squirrels (*Xerus erythropus*), and bats (*Scotophillus sp*) [1].

The identification of a sylvatic cycle of yellow fever virus (YFV) in southeastern Senegal [9,10], led to the establishment of a surveillance program that documented the recurrence of the epizootic amplifications by the isolation of virus from mosquitoes and the detection of antibodies in simian and human sera at 4–6 year intervals, during the rainy season [11]. A three year survey, consisting of monthly 25-hour human landing collections of the mosquito fauna in a transect from a forest-gallery to the nearest village, showed that only 4 species (*Ae. luteocephalus*, *Ae. vittatus*, *Ae. furcifer* and *Ae. taylori*) were attracted to humans [12]. Other species were considered as non-anthropophilic. That study also showed that these species were host-seeking in the evening and that the evening collection was representative of the entire mosquito fauna.

While sylvatic transmission of these viruses is relatively well characterized in Senegal, some aspects of the ecology of their vectors are still poorly understood. Notably, only one study has been devoted to the ecology of the larval stages of arbovirus vectors [13]. Moreover, that study was limited to a single gallery forest, while there are many other land cover classes in the area. We have recently reported the distribution and abundance of adult mosquitoes potentially involved in the sylvatic cycle of CHIKV in southeastern Senegal, as well as their levels of infection in the five most abundant land cover elements (forest, savanna, agriculture, barren and village) [14]. Potential vectors are found in each of the land cover classes, but *Ae. furcifer* was the only species that occured in all land cover types and also entered villages to feed on humans. Thus, this species is probably the most important bridge vector between forest circulation and human populations. However, the presence of a host seeking vector in a land cover type does not always mirror the distribution of its conspecific larvae.

In addition, some non-anthropophilic mosquito species, scarce or absent in a previous 25 hour human landing collection, and probably feeding mainly on animals that have been associated with YFV, DENV-2 and CHIKV, may have large populations in the Kédougou area [2]. Understanding the larval ecology of these vectors is of particular importance for monitoring and controlling the circulation and spillover of these sylvatic viruses. An investigation of these parameters will allow us to better understand the transmission cycles and therefore the epidemiology of these viruses. Furthermore, knowledge of larval vector ecology is a key factor in risk assessment and establishment of effective control strategies and tools, because the most effective method for controlling vector populations is to control the immature stages in their aquatic habitats before they emerge as adults.

The aim of this work was therefore to identify the larval habitats of potential arbovirus vectors in the Kédougou area and characterize their seasonal and spatial dynamics.

## Methods

### Study area

**Figure 1:**
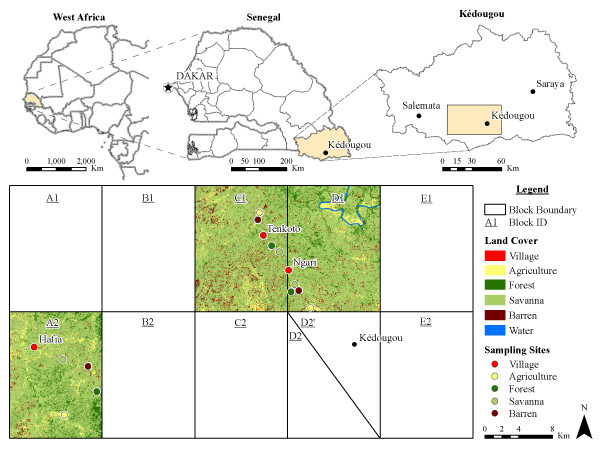
**Location of the study site.** The rectangle in the upper right map corresponds to the 1,650 Km^2^ divided in ten blocks (A2-E2) below. Data were collected in each of the five land covers (agriculture, barren, village, savannah and forest) in the blocks A2, C1 and D1.

Our study was undertaken in the Kédougou region (Figure 1) located in southeastern Senegal (12°33 N, 12°11 W). The annual rainfall ranges from 1200 to 1300 mm, with one rainy season between May and November, and the topography is hilly. Mean temperatures vary from 33–39.5°C during the year. Kédougou lies in a transition zone between the dry tropical forest and the savannah belt. A mosaic of forest, forest galleries and savannahs constitute the natural vegetation. The human population of the region is ca. 80,000 and is primarily rural (84%) with a low overall density of inhabitants (4/km^2^), mostly living in small, dispersed villages averaging 60 inhabitants. The fauna encountered is very diverse including three monkey species, the Guinea baboon (*Papio papio*), the patas monkey (*E. patas*), the African green monkey (*Cercopithecus sabaeus*), and one ape, the chimpanzee (*Pan troglodytes*).

### Larval habitat selection and classification

**Figure 2:**
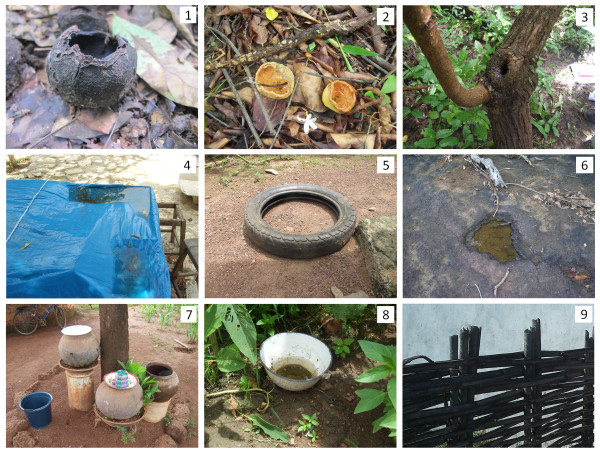
Immature mosquito microhabitats (1 = decaying fruit husks, 2 = fresh fruit husks, 3 = tree holes, 4 = puddles, 5= tires, 6 = rocks-holes, 7 = storage containers, 8 = discarded containers and 9 = bamboo holes) in Kédougou from June – December 2010.

An area of 1650 km^2^ (30 km in N-S direction; 55 km in E-W direction) of the Kédougou region (Figure 1) was divided into 10 blocks of roughly equal size. In each block, 5 different types of land cover, classified as forest, barren, savannah, agriculture and village, were defined by remote sensing and geospatial analyses, and one sampling site was chosen in each land cover class as described previously [14]. Based on previous data on the distribution and abundance of adult mosquitoes [14], blocks A2, C1 and D1 were chosen for this study of larval ecology. In a preliminary survey, all existing natural and artificial cavities or containers with the potential to hold water, in the different land cover classes, were recorded. These habitats were classified based on the origin, microhabitat, material and/or container type as decaying fruit husks, fresh fruit husks, puddles, tree holes, bamboo holes, tires, rocks-holes, discarded containers and storage containers (Figure 2). These habitat types were described as follows: 1) decaying fruit husks from the past year’s production of *Saba senegalensis* (Apocynacées), which are thick and rigid, with a globally hemispherical shape, black colored and of small size (less than 10 cm in diameter); 2) fresh fruit husks from the current year production of the same plant that are less rigid, yellow colored and hold different water quality (colored and acid) compared to the decaying fruit husk; 3) tree holes were rot and pan holes of different shapes and volume located from 0 to 2 m above the ground level; 4) puddles were temporary small water collections that formed on the ground after rainfall and in plastic sheets covering hen house roofs; 5) tires were used bicycle tires left outdoors within villages; 6) rocks holes were of irregular shapes, different sizes and were generally shallow, well exposed to solar radiation and located on lateritic carapaces; 7) storage containers were clay pots and plastic containers used to store potable water; 8) discarded containers were from human waste (broken clay pots, plastic bottles, bowls, metal box, used cans, etc.) and 9) bamboo holes were cut bamboo used as fences within villages.

### Sampling procedure

In each of the chosen blocks, the five land cover classes were surveyed and up to 30 of each of the 9 categorized microhabitats were examined for the presence of water and immature mosquitoes once per month from June, 2010 (just after the beginning of the rainy season) to January, 2011 (when no mosquito immature stages were found, thus the last mosquito larva was found in December, 2010). If larvae and/or pupae were present, the content of each hole was completely removed as follows. Small tree holes were emptied with a pipette of 10 ml volume composed of a rigid plastic tube with a rubber suction bulb fitted to one end and/or with a mouth aspirator composed by a 150 ml plastic pot with a cover connected to two flexible rubber tubes inserted through it. The longer one is inserted into the hole while the other is sucked for siphoning water out. Larger tree holes and rocks holes were sampled using either pipettes or small dippers (50–150 ml capacity). Bamboo holes were emptied by siphoning out the water with the mouth aspirator. Because all the larvae could not be removed by the initial siphoning, especially for tree holes [15], all holes were refilled with tap water and re-emptied until no larvae remained.

Hole contents were poured through a mesh net that retained all larval mosquito instars. The contents of discarded and storage containers were directly poured through a mesh net. For each sample (hole or container), the mesh net containing immature mosquitoes was submerged in tap water in a white plastic tray. *Toxorhynchites spp.* and *Cx. tigripes* larvae were removed from the sample to avoid predation of the other species. The content of the trays were placed in vials (different number depending on the quantity of larvae in the sample to avoid overcrowding and limit high mortalities of immature stages) labeled with a number corresponding to the microhabitat type, land cover class and date of collection. The holes were refilled to their original volume with tap water. No volumetric record of the sizes of microhabitats was done. Immature stages were reared to adults in a field insectary, fed with larvae from a colony reared especially for that predacious species, and with TetraMin Baby Fish Food **®** for the others. Larval mortalities were relatively low and were not recorded. Only some tree holes and storage containers were repeatedly sampled on successive months. The other microhabitats were chosen randomly among all those available in the land covers.

Adults that emerged from larval collections were identified according to the keys of Edwards [16], Ferrara *et al.*[17], Huang [18] and Jupp [19] for the culicines and by Diagne *et al*. [20] for the anophelines.

### Data analysis

Frequency of occurrence, expressed as the percentage of wet (water-holding) microhabitat that held immature mosquitoes, was calculated for the whole mosquito fauna and for each of the commonest species for each type of land cover class (macrohabitat) and microhabitat. Chi-square contingency tests were used to compare frequencies of occurrence of mosquitoes between types of micro and macrohabitats. Larval abundances (in the different micro and macrohabitats) were calculated as Williams’ Means (Mw) [21]. The Kruskal-Wallis *H* test was used to compare larval abundances between habitats and the Mann–Whitney *U* test was used between pairs of habitats when the Kruskal-Wallis test was found to be statistically significant or when only two habitats were being compared. Differences were considered significant when p < 0.05. The number of species collected and the specific dominance were calculated and compared between habitats using the biodiversity module of *Past* 2.14®. The C_7_ index of Cole [22] was used to evaluate the interspecific associations between the species representing more than 1% of the total mosquito fauna collected, and the statistical significance tested with the corrected χ^2^ according Pielou [23]. The Fisher’s exact test was used when one or more expected values were equal or less to five. All tests were conducted in *StatView 5.0* ®.

## Results

### Habitat positivity

**Figure 3:**
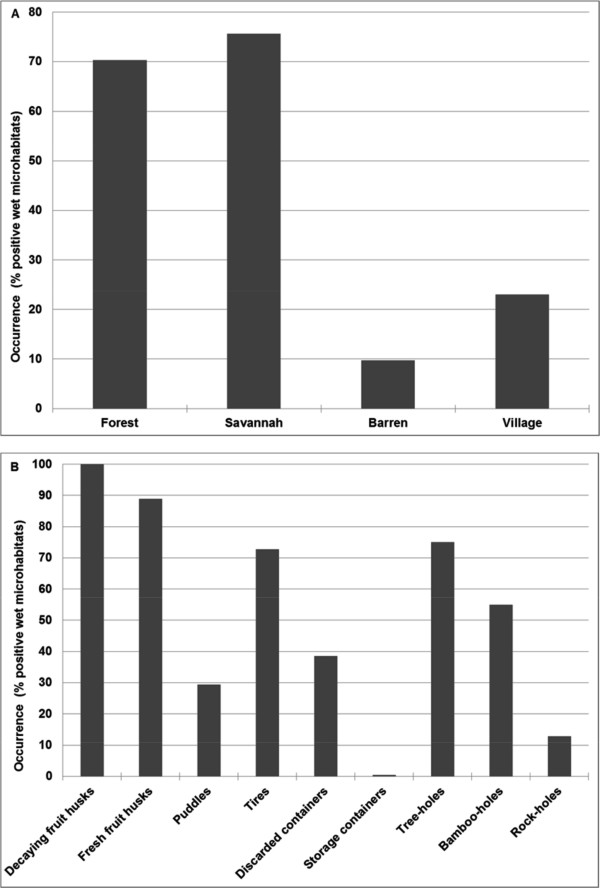
Occurrence of immature stages of common mosquito species in different land cover classes (A) and microhabitats (B), of sylvatic arbovirus foci, in Kédougou from June – December 2010.

**Figure 4:**
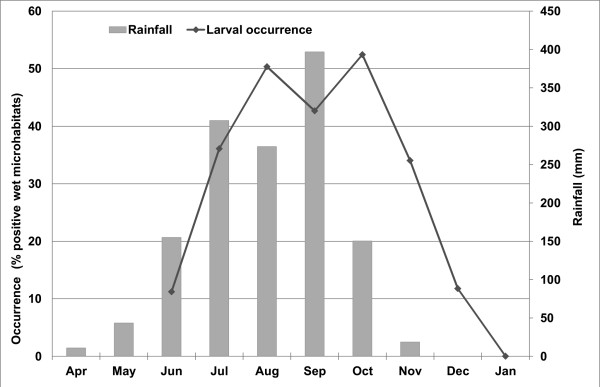
Seasonal occurrence of immature stages of common mosquito species in sylvatic arbovirus foci in Kédougou from June – December 2010.

Among the 2460 microhabitats examined, 1279 were wet, and 30.4% of these held at least one larva or pupa. Wet microhabitats were found in every land cover type except agricultural land. Larval occurrence (Figure 3) differed significantly among different land cover classes (χ^2^= 370.7; df = 3; p *<* 0.0001) and microhabitats (χ^2^= 549.6; df = 8; p *<* 0.0001). Microhabitats within forest and savannah land covers were most frequently occupied and were equally likely (χ^2^= 1.5; df = 1; p *=* 0.22) to be colonized by larvae of one or more species of mosquitoes. Regardless of the land cover type, immature mosquitoes were most often detected in decaying fruit husks, of which 100% contained at least one larva or pupa, followed by fresh fruit husks (89%), tree holes (75%) and tires (73%). The lowest frequencies of immature mosquito occurrence were observed in rocks holes (13%) and storage containers (0.4%). The percentage of infested wet microhabitats increased from June (just after the first rains) and peaked twice between August and October, after which it decreased (Figure 4).

### Species composition

Thirty-five species of mosquitoes in 7 genera were collected from natural and artificial water-holding microhabitats at our study sites from June to December, 2010 (Table 1). A total of 5121 mosquito adults emerged from larvae and pupae collected from forests (1858 specimens), savannahs (545), barren land (86) and villages (2632). *Aedes aegypti formosus* (the only sub species of *Ae. aegypti* present in the study area) was the dominant species, representing 30.2% of immature mosquitoes collected, followed by 12 other species, each comprising more than 1% of the mosquito fauna collected. The YFV, DENV-2 and CHIKV vectors included *Ae. vittatus* (7.9%), *Ae. luteocephalus* (5.7%), *Ae. taylori* (5.0%) and *Ae. furcifer* (1.3%). The dominant species varied according to the land cover and microhabitat investigated and was *Eretmapodites chrysogaster* (21.6%) in the forest, *Ae. vittatus* in the savannah (18.3%) and barren (65.1%) and *Ae. aegypti* in the village (53.3%). Among microhabitats (Table 2), the dominant species were *Ae. aegypti* in the storage containers (100% of the collected fauna), tires (96%), bamboo holes (90.6%) and discarded containers (51.4%), *Er. chrysogaster* in fresh fruit husks (64.7%) and decaying fruit husks (48.4%), *Ae. vittatus* in puddles (52.3) and rocks holes (48.3) and finally *Ae. luteocephalus* in tree holes with 20.2% of the collected fauna.

**Table 1:** Total number of mosquitoes collected as larvae in different land cover classes, within foci of sylvatic arboviruses in Kédougou from June – December 2010

**Species**	**Forest**		**Savannah**		**Barren**		**Village**		**Total per species**	
	**Nb**	**%**	**Nb**	**%**	**Nb**	**%**	**Nb**	**%**	**Nb**	**%**
*Aedes aegypti*	108	5.8	29	5.3	8	9.3	1402	53.3	1547	30.2
*Aedes africanus*	6	0.3		0.0		0.0		0.0	6	0.1
*Aedes argenteopunctatus*	1	0.1		0.0		0.0		0.0	1	0.0
*Aedes bromeliae*	4	0.2	1	0.2		0.0	34	1.3	39	0.8
*Aedes furcifer*	33	1.8	33	6.1		0.0		0.0	66	1.3
*Aedes hirsutus*		0.0		0.0		0.0	15	0.6	15	0.3
*Aedes longipalpis*	99	5.3	6	1.1		0.0	1	0.0	106	2.1
*Aedes luteocephalus*	180	9.7	94	17.2		0.0	18	0.7	292	5.7
*Aedes metallicus*	1	0.1		0.0		0.0		0.0	1	0.0
*Aedes minutus*	1	0.1		0.0		0.0		0.0	1	0.0
*Aedes neoafricatus*	1	0.1	1	0.2		0.0		0.0	2	0.0
*Aedes stokesi*	7	0.4		0.0		0.0		0.0	7	0.1
*Aedes taylori*	211	11.4	44	8.1		0.0		0.0	255	5.0
*Aedes unilineatus*	51	2.7	40	7.3		0.0	5	0.2	96	1.9
*Aedes vittatus*	149	8.0	100	18.3	56	65.1	98	3.7	403	7.9
*Anopheles coustani*	1	0.1		0.0		0.0		0.0	1	0.0
*Anopheles gambiae*	1	0.1		0.0	1	1.2	7	0.3	9	0.2
*Anopheles hancocki*		0.0		0.0	1	1.2		0.0	1	0.0
*Anopheles pretoriensis*	1	0.1		0.0	1	1.2		0.0	2	0.0
*Anopheles rufipes*	2	0.1		0.0	14	16.3		0.0	16	0.3
*Culex cinerus*	121	6.5	6	1.1		0.0	39	1.5	166	3.2
*Culex decens*	79	4.3	23	4.2		0.0	12	0.5	114	2.2
*Culex macfiei*	23	1.2	6	1.1		0.0	2	0.1	31	0.6
*Culex neavei*	1	0.1	1	0.2		0.0		0.0	2	0.0
*Culex nebulosus*	238	12.8	37	6.8		0.0	478	18.2	753	14.7
*Culex perfuscus*	73	3.9	8	1.5	1	1.2	75	2.8	157	3.1
*Culex tigripes*		0.0	5	0.9		0.0	33	1.3	38	0.7
*Culex tritaeniorhynchus*	1	0.1	4	0.7	1	1.2	373	14.2	379	7.4
*Eretmapodites chrysogaster*	401	21.6	99	18.2	3	3.5	26	1.0	529	10.3
*Eretmapodites oedipodius*		0.0		0.0		0.0	1	0.0	1	0.0
*Eretmapodites quinquevittatus*	4	0.2	2	0.4		0.0	10	0.4	16	0.3
*Ficalbia circumtestea*	10	0.5		0.0		0.0		0.0	10	0.2
*Toxorhynchites brevipalpis*	24	1.3	4	0.7		0.0	3	0.1	31	0.6
*Toxorhynchites lutescens*	3	0.2	2	0.4		0.0		0.0	5	0.1
*Uranotaenia mashonensis*	23	1.2		0.0		0.0		0.0	23	0.4
Total per land cover	1858	100	545	100	86	100	2632	100	5121	100

**Table 2:** Mosquitoes collected as larvae in different microhabitats, in sylvatic arbovirus foci in Kédougou from June – December 2010

**Species**	**Bamboo holes**		**Discarded containers**		**Decaying fruit husks**		**Fresh fruit husks**		**Puddles**		**Rock holes**		**Storage containers**		**Tree holes**		**Tires**	
	**Nb**	**%**	**Nb**	**%**	**Nb**	**%**	**Nb**	**%**	**Nb**	**%**	**Nb**	**%**	**Nb**	**%**	**Nb**	**%**	**Nb**	**%**
*Aedes aegypti*	48	90.6	1206	51.4	49	38.9	57	8.7	27	11.1	36	8.9	97	100	84	6.6	24	96
*Aedes africanus*	0	0.0	0	0.0	0	0.0	0	0.0	0	0.0	0	0.0	0	0	6	0.5	0	0
*Aedes argenteopunctatus*	0	0.0	0	0.0	0	0.0	0	0.0	0	0.0	0	0.0	0	0	1	0.1	0	0
*Aedes bromeliae*	0	0.0	31	1.3	1	0.8	2	0.3	0	0.0	0	0.0	0	0	2	0.2	1	4
*Aedes furcifer*	0	0.0	0	0.0	0	0.0	3	0.5	0	0.0	0	0.0	0	0	63	5.0	0	0
*Aedes hirsutus*	0	0.0	9	0.4	0	0.0	0	0.0	6	2.5	0	0.0	0	0	0	0.0	0	0
*Aedes longipalpis*	0	0.0	0	0.0	0	0.0	1	0.2	1	0.4	0	0.0	0	0	104	8.2	0	0
*Aedes luteocephalus*	0	0.0	14	0.6	3	2.4	16	2.4	3	1.2	0	0.0	0	0	256	20.2	0	0
*Aedes metallicus*	0	0.0	0	0.0	0	0.0	0	0.0	0	0.0	0	0.0	0	0	1	0.1	0	0
*Aedes minutus*	0	0.0	0	0.0	0	0.0	0	0.0	0	0.0	0	0.0	0	0	1	0.1	0	0
*Aedes neoafricatus*	0	0.0	0	0.0	0	0.0	0	0.0	0	0.0	0	0.0	0	0	2	0.2	0	0
*Aedes stokesi*	0	0.0	0	0.0	0	0.0	0	0.0	0	0.0	0	0.0	0	0	7	0.6	0	0
*Aedes taylori*	0	0.0	0	0.0	0	0.0	2	0.3	0	0.0	0	0.0	0	0	253	20.0	0	0
*Aedes unilineatus*	3	5.7	2	0.1	5	4.0	19	2.9	0	0.0	1	0.2	0	0	66	5.2	0	0
*Aedes vittatus*	0	0.0	69	2.9	0	0.0	3	0.5	127	52.3	195	48.3	0	0	9	0.7	0	0
*Anopheles coustani*	0	0.0	0	0.0	0	0.0	1	0.2	0	0.0	0	0.0	0	0	0	0.0	0	0
*Anopheles gambiae*	0	0.0	7	0.3	0	0.0	0	0.0	0	0.0	2	0.5	0	0	0	0.0	0	0
*Anopheles hancocki*	0	0.0	0	0.0	0	0.0	0	0.0	0	0.0	1	0.2	0	0	0	0.0	0	0
*Anopheles pretoriensis*	0	0.0	0	0.0	0	0.0	0	0.0	0	0.0	3	0.7	0	0	0	0.0	0	0
*Anopheles rufipes*	0	0.0	0	0.0	0	0.0	0	0.0	0	0.0	14	3.5	0	0	2	0.2	0	0
*Culex cinerus*	0	0.0	33	1.4	1	0.8	10	1.5	6	2.5	0	0.0	0	0	116	9.2	0	0
*Culex decens*	0	0.0	11	0.5	0	0.0	1	0.2	12	4.9	74	18.3	0	0	16	1.3	0	0
*Culex macfiei*	0	0.0	2	0.1	0	0.0	1	0.2	0	0.0	4	1.0	0	0	27	2.1	0	0
*Culex neavei*	0	0.0	0	0.0	0	0.0	0	0.0	0	0.0	1	0.2	0	0	1	0.1	0	0
*Culex nebulosus*	0	0.0	485	20.7	2	1.6	106	16.2	19	7.8	6	1.5	0	0	140	11.1	0	0
*Culex perfuscus*	0	0.0	53	2.3	0	0.0	1	0.2	23	9.5	37	9.2	0	0	43	3.4	0	0
*Culex tigripes*	0	0.0	32	1.4	0	0.0	0	0.0	1	0.4	0	0.0	0	0	5	0.4	0	0
*Culex tritaeniorhynchus*	0	0.0	355	15.1	2	1.6	2	0.3	18	7.4	3	0.7	0	0	1	0.1	0	0
*Eretmapodites chrysogaster*	2	3.8	24	1.0	61	48.4	424	64.7	0	0.0	4	1.0	0	0	14	1.1	0	0
*Eretmapodites oedipodius*	0	0.0	1	0.0	0	0.0	0	0.0	0	0.0	0	0.0	0	0	0	0.0	0	0
*Eretmapodites quinquevittatus*	0	0.0	10	0.4	2	1.6	3	0.5	0	0.0	0	0.0	0	0	1	0.1	0	0
*Ficalbia circumtestea*	0	0.0	0	0.0	0	0.0	0	0.0	0	0.0	0	0.0	0	0	10	0.8	0	0
*Toxorhynchites brevipalpis*	0	0.0	1	0.0	0	0.0	2	0.3	0	0.0	0	0.0	0	0	29	2.3	0	0
*Toxorhynchites lutescens*	0	0.0	0	0.0	0	0.0	1	0.2	0	0.0	0	0.0	0	0	6	0.5	0	0
*Uranotaenia mashonensis*	0	0.0	0	0.0	0	0.0	0	0.0	0	0.0	23	5.7	0	0	0	0.0	0	0
Total per microhabitat	53	100	2345	100	126	100	655	100	243	100	404	100	97	100	1266	100	25	100

### Species richness diversity and dominance

The species richness, diversity and dominances in the different land covers and microhabitats are presented in Table 3. The highest number of species and diversity were observed in the forest for the land covers (p ≤ 0.02) and the tree holes for the microhabitats (p ≤ 0.001) in this survey. Dominance in the forest was significantly less than in the other land covers (p ≤ 0.001). Among the microhabitats, tree holes had the lowest dominance (p ≤ 0.001). The following analyses take into account only the common species comprising more than 1% of the total mosquito fauna.

**Table 3:** Seasonal occurrence (% of positive wet containers) of immature stages of common mosquito species in different land covers and microhabitats, foci of sylvatic arbovirus, in Kédougou from June – December 2010

**Land covers**	**Species**	**Jun**	**Jul**	**Aug**	**Sep**	**Oct**	**Nov**	**Dec**	**Mean**
Forest	*Aedes aegypti*	10.8 (4)	16.4 (9)	10.3 (9)	3.3 (3)	4.8 (2)	14.3 (2)	0 (0)	8.7 (29)
	*Aedes furcifer*	5.4 (2)	7.3 (4)	7.7 (6)	6.7 (6)	2.4 (1)	0 (0)	0 (0)	3.9 (13)
	*Aedes longipalpis*	10.8 (4)	10.9 (6)	10.2 (8)	4.4 (4)	7.1 (3)	0 (0)	0 (0)	7.5 (25)
	*Aedes luteocephalus*	16.2 (6)	21.8 (12)	9.0 (7)	8.9 (8)	7.1 (3)	0 (0)	5.9 (1)	10.8 (36)
	*Aedes taylori*	16.2 (6)	25.4 (14)	7.7 (6)	7.8 (7)	2.4 (1)	0 (0)	0 (0)	10.2 (34)
	*Aedes unilineatus*	13.5 (5)	10.9 (6)	5.1 (4)	5.6 (5)	0 (0)	7.1 (1)	5.9 (1)	6.3 (21)
	*Aedes vittatus*	13.5 (5)	3.6 (2)	6.4 (5)	1.1 (1)	4.8 (2)	14.3 (2)	0 (0)	5.1 (17)
	*Culex cinerus*	13.5 (5)	36.4 (20)	5.1 (4)	0 (0)	0 (0)	0 (0)	0 (0)	8.7 (29)
	*Culex decens*	5.4 (2)	1.8 (1)	3.8 (3)	1.1 (1)	9.5 (4)	0 (0)	0 (0)	3.3 (11)
	*Culex nebulosus*	16.2 (6)	27.3 (15)	10.2 (8)	2.2 (2)	0 (0)	7.1 (1)	5.9 (1)	9.6 (32)
	*Culex perfuscus*	5.4 (2)	1.8 (1)	0 (0)	1.1 (1)	9.5 (4)	14.3 (2)	0 (0)	3.0 (10)
	*Culex tritaeniorhynchus*	0 (0)	0 (0)	0 (0)	0 (0)	2.4 (1)	0 (0)	0 (0)	0.3 (1)
	*Eretmapodites chrysogaster*	2.7 (1)	14.5 (8)	46.1 (36)	15.5 (14)	16.7 (7)	0 (0)	0 (0)	19.8 (66)
Savannah	*Aedes aegypti*	0 (0)	24 (6)	29.5 (13)	10.7 (7)	11.1 (1)	na	0 (0)	18.2 (27)
	*Aedes furcifer*	0 (0)	4 (1)	15.9 (7)	10.7 (7)	11.1 (1)	na	0 (0)	10.8 (16)
	*Aedes longipalpis*	0 (0)	0 (0)	2.3 (1)	1.5 (1)	0 (0)	na	0 (0)	1.4 (2)
	*Aedes luteocephalus*	0 (0)	12 (3)	18.2 (8)	17.9 (12)	22.2 (2)	na	0 (0)	16.9 (25)
	*Aedes taylori*	0 (0)	12 (3)	15.9 (7)	6.0 (4)	0 (0)	na	0 (0)	9.5 (14)
	*Aedes unilineatus*	0 (0)	8 (2)	13.6 (6)	9.0 (6)	11.1 (1)	na	0 (0)	10.1 (15)
	*Aedes vittatus*	50 (1)	4 (1)	4.5 (2)	0 (0)	0 (0)	na	0 (0)	2.7 (4)
	*Culex cinerus*	0 (0)	4 (1)	4.5 (2)	0 (0)	0 (0)	na	0 (0)	2.0 (3)
	*Culex decens*	0 (0)	0 (0)	9.1 (4)	0 (0)	0 (0)	na	0 (0)	2.7 (4)
	*Culex nebulosus*	0 (0)	20 (5)	9.1 (4)	1.5 (1)	0 (0)	na	0 (0)	6.8 (10)
	*Culex perfuscus*	0 (0)	0 (0)	6.8 (3)	3.0 (2)	0 (0)	na	0 (0)	3.4 (5)
	*Culex tritaeniorhynchus*	0 (0)	4 (1)	0 (0)	1.5 (1)	0 (0)	na	0 (0)	1.4 (2)
	*Eretmapodites chrysogaster*	0 (0)	24 (6)	36.4 (16)	9.0 (6)	0 (0)	na	0 (0)	18.9 (28)
Barren	*Aedes aegypti*	0 (0)	1.7 (1)	0 (0)	0 (0)	0 (0)	na	na	0.4 (1)
	*Aedes vittatus*	0 (0)	6.7 (4)	5.0 (3)	1.7 (1)	8.0 (2)	na	na	4.3 (10)
	*Culex nebulosus*	0 (0)	1.7 (1)	1.7 (1)	0 (0)	0 (0)	na	na	0.9 (2)
	*Culex perfuscus*	0 (0)	0 (0)	0 (0)	0 (0)	4.0 (1)	na	na	0.4 (1)
	*Culex tritaeniorhynchus*	0 (0)	1.7 (1)	0 (0)	0 (0)	8.0 (2)	na	na	1.3 (3)
	*Eretmapodites chrysogaster*	0 (0)	0 (0)	0 (0)	0 (0)	4.0 (1)	na	na	0.4 (1)
Village	*Aedes aegypti*	5.1 (4)	7.8 (7)	23.1 (25)	26.0 (32)	11.9 (8)	0 (0)	0 (0)	13.9 (76)
	*Aedes longipalpis*	1.3 (1)	0 (0)	0 (0)	0 (0)	0 (0)	0 (0)	0 (0)	0.2 (1)
	*Aedes luteocephalus*	0 (0)	4.4 (4)	1.8 (2)	4.1 (5)	1.5 (1)	0 (0)	0 (0)	2.2 (12)
	*Aedes unilineatus*	0 (0)	0 (0)	0.9 (1)	2.4 (3)	0 (0)	0 (0)	0 (0)	0.7 (4)
	*Aedes vittatus*	2.5 (2)	3.3 (3)	4.6 (5)	0.8 (1)	1.5 (1)	0 (0)	0 (0)	2.2 (12)
	*Culex cinerus*	2.5 (2)	2.2 (2)	2.8 (3)	0 (0)	0 (0)	0 (0)	0 (0)	1.3 (7)
	*Culex decens*	0 (0)	1.1 (1)	1.8 (2)	1.6 (2)	0 (0)	0 (0)	0 (0)	0.9 (5)
	*Culex nebulosus*	3.8 (3)	3.3 (3)	5.5 (6)	3.2 (4)	4.5 (3)	2.8 (1)	2.3 (1)	3.9 (21)
	*Culex perfuscus*	1.3 (1)	3.3 (3)	3.7 (4)	0 (0)	1.5 (1)	0 (0)	0 (0)	1.7 (9)
	*Culex tritaeniorhynchus*	0 (0)	12.2 (11)	2.8 (3)	5.7 (7)	4.5 (3)	2.8 (1)	0 (0)	4.6 (25)
	*Eretmapodites chrysogaster*	0 (0)	0 (0)	1.7 (1)	2.4 (3)	4.5 (3)	0 (0)	2.3 (1)	1.5 (8)
Microhabitat									
Decaying fruit husks	*Aedes aegypti*	na	60.7 (4)	33.3 (3)	na	na	na	na	46.7 (7)
	*Aedes luteocephalus*	na	0.0 (0)	11.1 (1)	na	na	na	na	6.7 (1)
	*Aedes unilineatus*	na	16.7 (1)	33.3 (3)	na	na	na	na	26.7 (4)
	*Culex cinerus*	na	16.7 (1)	0.0 (0)	na	na	na	na	6.7 (1)
	*Culex nebulosus*	na	33.3 (2)	0.0 (0)	na	na	na	na	13.3 (2)
	*Culex tritaeniorhynchus*	na	16.7 (1)	0.0 (0)	na	na	na	na	6.7 (1)
	*Eretmapodites chrysogaster*	na	50.0 (3)	100 (9)	na	na	na	na	80.0 (12)
Fresh fruit husks	*Aedes aegypti*	0.0 (0)	22.2 (4)	30.9 (13)	2.1 (1)	6.7 (1)	na	na	15.0 (19)
	*Aedes furcifer*	0.0 (0)	5.6 (1)	2.4 (1)	2.1 (1)	0.0 (0)	na	na	2.4 (3)
	*Aedes longipalpis*	0.0 (0)	0.0 (0)	2.4 (1)	0.0 (0)	0.0 (0)	na	na	0.8 (1)
	*Aedes luteocephalus*	0.0 (0)	0.0 (0)	4.8 (2)	4.2 (2)	6.7 (1)	na	na	3.9 (5)
	*Aedes taylori*	0.0 (0)	5.6 (1)	0.0 (0)	2.1 (1)	0.0 (0)	na	na	1.6 (2)
	*Aedes unilineatus*	0.0 (0)	0.0 (0)	7.1 (3)	4.2 (2)	0.0 (0)	na	na	3.9 (5)
	*Aedes vittatus*	0.0 (0)	0.0 (0)	4.8 (2)	0.0 (0)	0.0 (0)	na	na	1.6 (2)
	*Culex cinerus*	0.0 (0)	16.7 (3)	0.0 (0)	0.0 (0)	0.0 (0)	na	na	2.4 (3)
	*Culex decens*	0.0 (0)	0.0 (0)	2.4 (1)	0.0 (0)	0.0 (0)	na	na	0.8 (1)
	*Culex nebulosus*	0.0 (0)	27.8 (5)	4.8 (2)	0.0 (0)	0.0 (0)	na	na	5.5 (7)
	*Culex perfuscus*	0.0 (0)	0.0 (0)	0.0 (0)	0.0 (0)	6.7 (1)	na	na	0.8 (1)
	*Culex tritaeniorhynchus*	0.0 (0)	0.0 (0)	0.0 (0)	2.1 (1)	0.0 (0)	na	na	0.8 (1)
	*Eretmapodites chrysogaster*	0.0 (0)	55.6 (10)	92.8 (39)	38.3 (18)	40 (6)	na	na	57.5 (73)
Puddles	*Aedes aegypti*	16.7 (1)	22.2 (2)	18.2 (2)	0 (0)	100 (1)	na	0.0 (0)	11.8 (6)
	*Aedes longipalpis*	16.7 (1)	0.0 (0)	0.0 (0)	0.0 (0)	0.0 (0)	na	0.0 (0)	2.0 (1)
	*Aedes luteocephalus*	0.0 (0)	11.1 (1)	9.1 (1)	4.8 (1)	0.0 (0)	na	0.0 (0)	5.9 (3)
	*Aedes vittatus*	16.7 (1)	33.3 (3)	27.3 (3)	0.0 (0)	0.0 (0)	na	0.0 (0)	13.7 (7)
	*Culex cinerus*	16.7 (1)	11.1 (1)	9.1 (1)	0.0 (0)	0.0 (0)	na	0.0 (0)	5.9 (3)
	*Culex decens*	0.0 (0)	0.0 (0)	9.1 (1)	4.8 (1)	0.0 (0)	na	0.0 (0)	3.9 (2)
	*Culex nebulosus*	16.7 (1)	33.3 (3)	9.1 (1)	0.0 (0)	100 (1)	na	0.0 (0)	11.8 (6)
	*Culex perfuscus*	0.0 (0)	22.2 (2)	9.1 (1)	4.8 (1)	0.0 (0)	na	0.0 (0)	7.8 (4)
	*Culex tritaeniorhynchus*	0.0 (0)	33.3 (3)	0.0 (0)	0.0 (0)	0.0 (0)	na	0.0 (0)	5.9 (3)
Discarded containers	*Aedes aegypti*	7.3 (3)	9.7 (4)	38.5 (20)	37.1 (23)	21.9 (7)	0.0 (0)	0.0 (0)	23.7 (57)
	*Aedes luteocephalus*	0.0 (0)	7.3 (3)	1.9 (1)	4.8 (3)	3.1 (1)	0.0 (0)	10 (1)	3.7 (9)
	*Aedes unilineatus*	0.0 (0)	0.0 (0)	1.9 (1)	1.6 (1)	0.0 (0)	0.0 (0)	0.0 (0)	0.8 (2)
	*Aedes vittatus*	2.4 (1)	2.4 (1)	5.8 (3)	1.6 (1)	3.1 (1)	0.0 (0)	0.0 (0)	2.9 (7)
	*Culex cinerus*	2.4 (1)	2.4 (1)	3.8 (2)	0.0 (0)	0.0 (0)	0.0 (0)	0.0 (0)	1.7 (4)
	*Culex decens*	0.0 (0)	2.4 (1)	1.9 (1)	3.2 (2)	0.0 (0)	0.0 (0)	0.0 (0)	1.7 (4)
	*Culex nebulosus*	4.9 (2)	2.4 (1)	9.6 (5)	4.8 (3)	6.2 (2)	33.3 (1)	20 (2)	6.6 (16)
	*Culex perfuscus*	2.4 (1)	2.4 (1)	5.8 (3)	0.0 (0)	3.1 (1)	0.0 (0)	10 (1)	2.9 (7)
	*Culex tritaeniorhynchus*	0.0 (0)	19.5 (8)	5.8 (3)	11.3 (7)	9.4 (3)	33.3 (1)	0.0 (0)	9.1 (22)
	*Eretmapodites chrysogaster*	0.0 (0)	0.0 (0)	1.9 (1)	4.8 (3)	6.2 (2)	0.0 (0)	10 (1)	2.9 (7)
Storage containers	*Aedes aegypti*	0.0 (0)	2.5 (1)	0.0 (0)	0.0 (0)	0.0 (0)	0.0 (0)	0.0 (0)	0.4 (1)
Tree holes	*Aedes aegypti*	17.4 (4)	15.5 (7)	8.2 (5)	11.6 (10)	6.7 (2)	0.0 (0)	0.0 (0)	10.9 (28)
	*Aedes furcifer*	8.7 (2)	8.9 (4)	19.7 (12)	13.9 (12)	6.7 (2)	0.0 (0)	0.0 (0)	12.5 (32)
	*Aedes longipalpis*	17.4 (4)	13.3 (6)	13.1 (8)	5.8 (5)	10 (3)	0.0 (0)	0.0 (0)	10.2 (26)
	*Aedes luteocephalus*	26.1 (6)	33.3 (15)	19.7 (12)	22.1 (19)	13.3 (4)	0.0 (0)	0.0 (0)	21.9 (56)
	*Aedes taylori*	26.1 (6)	35.5 (16)	21.3 (13)	11.6 (10)	3.3 (1)	0.0 (0)	0.0 (0)	18.0 (46)
	*Aedes unilineatus*	17.4 (4)	15.5 (7)	6.5 (4)	10.5 (9)	3.3 (1)	25 (1)	14.3 (1)	10.5 (27)
	*Aedes vittatus*	13.0 (3)	2.2 (1)	3.3 (2)	1.2 (1)	3.3 (1)	0.0 (0)	0.0 (0)	3.1 (8)
	*Culex cinerus*	21.7 (5)	37.8 (17)	9.8 (6)	0.0 (0)	0.0 (0)	0.0 (0)	0.0 (0)	10.9 (28)
	*Culex decens*	4.3 (1)	0.0 (0)	6.5 (4)	0.0 (0)	0.0 (0)	0.0 (0)	0.0 (0)	2.0 (5)
	*Culex nebulosus*	26.1 (6)	26.7 (12)	16.4 (10)	4.6 (4)	0.0 (0)	0.0 (0)	0.0 (0)	12.5 (32)
	*Culex perfuscus*	0.0 (0)	0.0 (0)	0.0 (0)	2.3 (2)	0.0 (0)	0.0 (0)	0.0 (0)	0.8 (2)
	*Culex tritaeniorhynchus*	0.0 (0)	0.0 (0)	0.0 (0)	0.0 (0)	3.3 (1)	0.0 (0)	0.0 (0)	0.4 (1)
	*Eretmapodites chrysogaster*	4.3 (1)	2.2 (1)	8.2 (5)	2.3 (2)	3.3 (1)	0.0 (0)	0.0 (0)	3.9 (10)
Bamboo holes	*Aedes aegypti*	na	na	42.8 (3)	55.5 (5)	0.0 (0)	na	na	40.8 (8)
	*Aedes unilineatus*	na	na	0.0 (0)	22.2 (2)	0.0 (0)	na	na	10.0 (2)
	*Eretmapodites chrysogaster*	na	na	0.0 (0)	0.0 (0)	25 (1)	na	na	5.0 (1)
Rock holes	*Aedes aegypti*	0.0 (0)	1.4 (1)	0.0 (0)	0.0 (0)	0.0 (0)	0.0 (0)	na	0.3 (1)
	*Aedes unilineatus*	1.4 (1)	0.0 (0)	0.0 (0)	0.0 (0)	0.0 (0)	0.0 (0)	na	0.3 (1)
	*Aedes vittatus*	2.8 (2)	7.1 (5)	7.1 (5)	1.4 (1)	10.3 (3)	20 (2)	na	5.6 (18)
	*Culex decens*	1.4 (1)	1.4 (1)	1.4 (1)	0.0 (0)	0.0 (0)	40 (4)	na	2.2 (7)
	*Culex nebulosus*	0.0 (0)	1.4 (1)	1.4 (1)	0.0 (0)	0.0 (0)	10 (1)	na	0.9 (3)
	*Culex perfuscus*	1.4 (1)	1.4 (1)	0.0 (0)	0.0 (0)	3.4 (1)	20 (2)	na	1.6 (5)
	*Culex tritaeniorhynchus*	0.0 (0)	1.4 (1)	0.0 (0)	0.0 (0)	6.9 (2)	0.0 (0)	na	0.9 (3)
	*Eretmapodites chrysogaster*	0.0 (0)	0.0 (0)	1.4 (1)	0.0 (0)	3.4 (1)	0.0 (0)	na	0.6 (2)
Tires	*Ae. aegypti*	0.0 (0)	0.0 (0)	0.0 (0)	75 (3)	0.0 (0)	na	0.0 (0)	27.3 (3)

Number of microhabitats positive for each data entry is in parentheses.

### Larval frequencies of occurrence and dynamics of common species

Larval occurrence in the different land cover classes and microhabitat types varied by species (Table 4). *Aedes aegypti*, *Cx. nebulosus*, *Cx. perfuscus*, *Cx. tritaeniorhynchus*, *Er. chrysogster* and *Ae. vittatus* were collected from all positive land cover classes, whereas *Ae. furcifer* and *Ae. taylori* were found only in forests and savannahs; among the other common species in forests, savannahs and villages (Table 2). *Aedes aegypti* was the only common species collected in all the 9 microhabitats encountered in this study, whereas *Ae. furcifer and Ae. taylori were present in fresh fruit husks and tree holes* (Table 3); the other common species were more evenly distributed.

**Table 4:** Abundance of immature stages of common mosquito species in different microhabitats, in sylvatic arbovirus foci, in Kédougou from June – December 2010

**Species**	**Decaying fruit husks**	**Fresh fruit husks**	**Puddles**	**Discarded containers**	**Tree holes**	**Bamboo holes**	**Rock holes**	**Tires**	**Storage containers**
*Aedes aegypti*	1.37^a^	0.2^b^	0.14^b^	0.77^a,b^	0.14^b^	0.88^a,b^	0.02^c^	0.6^a,b^	0.02^c^
*Aedes furcifer*		0.02^b^			0.13^a^				
*Aedes longipalpis*		0.005^b^	0.01^a,b^		0.14^a^				
*Aedes luteocephalus*	0.1^a,b^	0.05^b^	0.04^b^	0.03^b^	0.35^a^				
*Aedes taylori*		0.01^b^			0.3^a^				
*Aedes unilineatus*	0.23^b^	0.06^b^		0.006^c^	0.12^a^	0.09^a,b^	0.002^c^		
*Aedes vittatus*		0.014^b^	0.44^a^	0.05^b^	0.02^b^		0.11^b^		
*Culex cinerus*	0.05^a,b^	0.03^b^	0.06^a,b^	0.03^b^	0.16^a^				
*Culex decens*		0.005	0.07	0.02	0.02		0.04		
*Culex nebulosus*	0.1^a^	0.1^a^	0.15^a^	0.16^a^	0.19^a^		0.009^b^		
*Culex perfuscus*		0.005	0.15	0.04	0.05		0.03		
*Culex tritaeniorhynchus*	0.07^a^	0.009^b^	0.1^a^	0.2^a^	0.003^b^		0.006^b^		
*Eretmapodites chrysogaster*	2.1^a^	1.5^a^		0.04^b^	0.03^b^	0.06^b^	0.006^c^		

Letters indicate the results of paired Mann–Whitney test when the Kruskal-Wallis test was found statistically significant or when only two habitats were being compared. Groups that do not share a letter are significantly different (P < 0.05).

*Aedes furcifer* immature stages were significantly more likely to be encountered in savannahs (χ^2^= 3.96; df = 1; p *=* 0.04), and *Ae. longipalpis* and *Cx. cinerus* in forests (p *≤* 0.007), whereas some others species (*Ae. luteocephalus*, *Ae. unilineatus*, *Cx. decens*, *Er. Chrysogaster* and *Ae. taylori*) were found almost equally in forests and savannahs (p *≥* 0.06). *Aedes aegypti* and *Cx. tritaeniorhychus* were equally likely to occur in savannahs and villages (p *≥* 0.07). *Aedes vittatus* and *Cx. nebulosus*, in contrast, occurred at statistically comparable frequencies in all four land cover classes (p *≥* 0.1).

Not all of the species analyzed had comparable frequencies among microhabitats. *Ae. furcifer, Ae. longipalpis* and *Ae. taylori* were most frequent in tree holes (p *≤* 0.001), *Ae. vittatus* and *Cx. perfuscus* in puddles (p *≤* 0.0002)*.* The other species were more evenly distributed and were detected in higher and statistically comparable frequencies in different combinations of microhabitats. Indeed, *Ae. aegypti* was detected in higher and comparable frequencies in decaying fruit husks, bamboo holes, tires and discarded containers, *Ae. luteocephalus* in tree holes and decaying fruit husks, *Ae. unilineatus* in tree holes, bamboo holes and decaying fruit husks, *Er. Chrysogaster* in decaying fruit husks and fresh fruit husks, *Cx. cinerus* in decaying fruit husks, tree holes and puddles, *Cx. nebulosus* in decaying fruit husks, fresh fruit husks, puddles, discarded containers and tree holes, *Cx. tritaeniorhychus* in decaying fruit husks, puddles and discarded containers and finally *Cx. decens* in puddles, discarded containers, tree holes and rock holes (p *≥* 0.09).

Immature stages of twelve of the thirteen most common species appeared for the first time in June in the forest land cover for *Ae. furcifer*, *Ae. taylori*, *Ae. luteocephalus*, *Ae. unilineatus*, *Cx. decens* and *Er. chrysogaster*, in the forest and the savannah for *Ae. vittatus* and in forest and village for *Ae. aegypti*, *Ae. longipalpis*, *Cx. cinerus*, *Cx. nebulosus* and *Cx. perfuscus* (Table 4). *Culex tritaeniorhynchus* appeared for the first time only in July in the village, barren and savannah land covers. These species were collected for the last time in December for *Ae. luteocephalus*, *Ae. unilineatus* and *Cx. nebulosus*, in November for *Ae. aegypti*, *Ae. vittatus* and *Cx. perfuscus* and in October for the other species.

### Larval abundance by species

**Figure 5:**
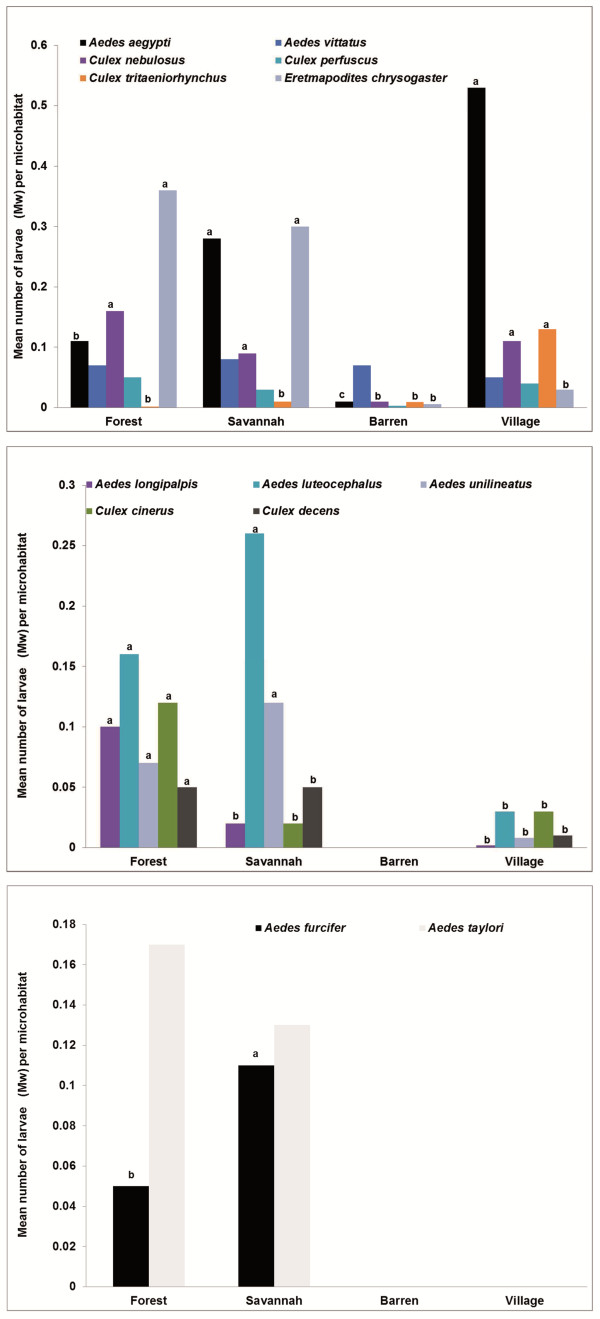
**Abundance of immature stages (larvae/ wet container) of common mosquito species in different land cover classes, foci of sylvatic arbovirus, in Kédougou from June – December 2010.** Letters indicate the results of a paired Mann–Whitney test when the Kruskal-Wallis test was found statistically significant or when only two habitats were being compared. Groups that do not share a letter are significantly different (P < 0.05).

The abundance of each species was compared among land cover or infested microhabitat types where they occurred. The analysis revealed statistically significant variations in larval abundances in the different land cover classes for all species except *Ae. taylori* (*U* = 24844, Z = −0.3, p = 0.8) and *Ae. vittatus* (*H* = 5.9, p = 0.1) (Figure 5). *Aedes aegypti* larvae were most abundant in villages and savannahs, *Ae. luteocephalus*, *Ae. unilineatus* and *Er. chrysogaster* in savannahs and forests, *Cx. nebulosus* in savannahs, forests and villages, *Ae. longipalpis*, *Cx. cinerus* and *Cx. decens* in forests (p *≥* 0.08) and *Ae. furcifer* in savannahs (p *<* 0.05).

Among microhabitats, there were statistically significant variations in larval abundances for all the species except *Cx. decens* (*H* = 2.2, p = 0.7) and *Cx. perfuscus* (*H* = 9.4, p = 0.05) (Table 5). *Aedes furcifer* (*U* = 17923.5, Z = −3.3, p = 0.001), *Ae. taylori* (*U* = 18952.5, Z = −4.6, p < 0.0001) and *Cx. nebulosus* (p *<* 0.05) were most abundant in tree holes, *Ae. vittatus* in puddles and tree holes*, Ae. luteocephalus* in tree holes and decaying fruit husks, *Ae. longipalpis* in tree holes and puddles, *Ae. unilineatus* in decaying fruit husks, tree holes and bamboo holes, *Er. chrysogaster* in decaying fruit husks and fresh fruit husks, *Cx. cinerus* in tree holes, decaying fruit husks and puddles, *Cx. tritaeniorhynchus* in discarded containers, decaying fruit husks and puddles and finally *Ae. aegypti* in decaying fruit husks, discarded containers, bamboo holes and tires (p *≥* 0.07).

**Table 5:** Richness and dominance of mosquito species in different land covers and microhabitats, within foci of sylvatic arbovirus, in Kédougou from June – December 2010

**Macrohabitat**	**Richness**	**Dominance**	**Shannon diversity**
Forest	31^a^	0.11^c^	2.52^a^
Savannah	21^b^	0.12^b^	2.36^b^
Barren	11^b^	0.37^a^	1.47^c^
Village	19^b^	0.34^a^	1.52^c^
Microhabitat			
Decaying fruit husks	9^c^	0.39^b^	1.21^d^
Fresh fruit husks	19^b^	0.4^b^	1.25^d^
Puddles	11^c^	0.31^c^	1.63^b^
Discarded containers	18^b^	0.33^c^	1.5^c^
Tree holes	28^a^	0.12^d^	2.42^a^
Bamboo holes	3^d^	0.82^a^	0.38^e^
Rock holes	15^b^	0.29^c^	1.67^b^
Tires	2^d^	0.92^a^	0.17^e^
Storage containers	1^d^	1^a^	0^f^

Groups that do not share a letter are significantly different (Bootstrap; P < 0.05).

### Interspecific association of the common species

The 66 pairings between the 12 most common species (Table 6) revealed 15 significant associations (10 positive and 5 negative associations). The highest positive and significant associations were between *Ae. luteocephalus* and *Ae. furcifer*, *Ae. taylori* and *Ae. furcifer*, and *Ae. luteocephalus* and *Ae. longipalpis*. All the five significant negative associations involved *Er. Chrysogaster* with *Ae. luteocephalus*, *Ae. taylori*, *Ae. longipalpis*, *Cx. cinerus* and *Cx. decens*.

**Table 6:** **Coefficients of interspecific association (C**_**7**_**) for the most common mosquito species in foci of sylvatic arbovirus in Kédougou from June – December 2010**

	***aegypti***	***furcifer***	***longi***	***luteo***	***taylori***	***unilin***	***vittatus***	***cinerus***	***decens***	***nebulo***	***perfus***	***chryso***
*furcifer*	0.06											
*longi*	0.05	0.1										
*luteo*	0.2	0.5***	0.1									
*taylori*	0.07	0.4***	0.4***	0.4***								
*unilin*	0.08	−0.1	−0.4	0.3**	0.04							
*vittatus*	−0.3	0.01	−0.2	−0.7	−0.4	−0.06						
*cinerus*	0.1	−0.04	0.2*	0.04	0.3***	−0.4	0.01					
*decens*	0.1	−0.1	−1	−01	−0.5	−0.3	0.09	0.02				
*nebulo*	0.3*	0.03	0.06	0.08	0.1	0.1	−0.7	0.4***	−0.03			
*perfus*	0.4	−1	−1	−1	−0.5	−0.2	0.1	−1	0.5***	−1		
*chryso*	0.3	−0.3	−0.7**	−0.6**	−0.5**	−0.1	−0.4	−0.6**	−1*	−0.3	−0.7	
*tritaenio*	−0.2	−1	−1	−1	−1	−1	−1	−1	−1	−1	−1	1

*Aegypti = Ae. aegypti, furcifer = Ae. furcifer, longi = Ae. longipalpis, luteo = Ae. luteocephalus, taylori = Ae. taylori, unilin = Ae. unilineatus, vittatus = Ae. vittatus, cinerus = Cx. cinerus, decens = Cx. decens, nebulo = Cx. nebulosus, perfus = Cx. perfuscus* and *tritaenio = Cx. tritaeniorynchus*. Levels of significance for χ^2^: *p < 0.05, **p < 0.01,*** p < 0.001.

## Discussion

Our larval collections yielded 15 more mosquito species than the only previous larval survey in the Kédougou region [13]. This higher number of species is likely due to the greater number of forested sites and more diverse land cover classes that we investigated. Indeed, the previous study [13] focused only on a single gallery forest, while we sampled forests, savannahs, barren areas and villages.

Only about one-third of the available water-filled microhabitats were occupied by immature mosquitoes, suggesting the possibility that gravid females choose their oviposition sites carefully. Similar findings have been reported by previous investigations [24-26]. Among others, the location of the microhabitat, its color, the chemical composition of the water, the quality and availability of food may be important factors determining mosquito frequencies of occurrence in water-filled microhabitats [27]. Recently, Wong *et al*. [28] also documented that *Ae. aegypti* exhibits strong conspecific attraction during oviposition site selection.

Larvae were detected between June and October-December, depending on the species. This pattern indicated clearly that rainfall is a key factor in larval ecology in Kédougou. Indeed, all the natural and artificial microhabitats used as larval habitats were filled by rainfall.

It was noteworthy that immature *Ae. furcifer* and *Ae. taylori*, two of the main YF, DENV-2 and CHIKV vectors, were collected in a very restricted range of habitats in contrast to *Ae. aegypti* and *Ae. vittatus*, which were found in a wide range of habitats. *Aedes luteocephalus* had a slightly different pattern of larval distribution. It was collected mainly in forests and savannahs within tree holes and fruit husks but was also collected in villages, albeit at a lower frequency.

The positive associations between *Ae. furcifer, Ae. luteocephalus* and *Ae. taylori* suggest that their gravid females follow the same ovipositional stimuli and illustrate their common larval preference for tree holes that probably have attractive physical and chemical elements for these species. Negative associations between *Er. Chrysogaster* and some species may be due, at least in part, to different ovipositional stimuli or to kairomonal repellents emitted by this species against the others. The same chemical product may be a stimulus to its conspecific.

Like the observation of Bang *et al*. [29] in Nigeria, but contrary to what was observed in East Africa [30], we detected a high diversity of mosquito larvae in domestic environments, although it was lower than the diversity in the forest. The higher species richness in tree holes may be due to their higher stability and trophic richness compared to the other microhabitats. Indeed, tree holes retained water for longer periods of time than the other microhabitats, which made them ideal larval sites for more species. Immatures in the tree holes may also be better protected against flushing during heavy rains.

The high occurrence of larval *Ae. aegypti formosus* in villages in the Kédougou region was not expected, because this subspecies is reported to undergo larval development in the forest, specifically in tree holes, rock holes and fruit husks [31]. However, its larvae have been found indoors in villages in Nigeria [32] and Gabon [33]. Discarded containers were among the main habitats for immature *Ae. aegypti* in villages in our study, suggesting a strong impact of human activities on the distribution of this species. The high container index of *Ae. aegypti* may suggest that the area is at high risk of YFV and other sylvatic arbovirus epidemics. However, human landing data [2,12,14] indicate that this species is minimally attracted to humans in the area. Virus isolations and vector competence studies also indicate that this species is rarely associated with arbovirus infection and has a low susceptibility to DENV-2 [2,14,34,35]. Thus, *Ae. aegypti* larval indices should be interpreted with caution in epidemiological risk evaluation for some rural areas of Africa because peridomestic larval habitats may be occupied by a highly zoophilic population of *Ae. aegypti formosus*. Despite a high degree of water storage, making many containers available as potential larval habitats, only one clay pot was found occupied by *Ae. aegypti formosus* during our study. This may indicate the sylvatic nature of this species and/or that adaption to peridomestic environments is ongoing. Our data thus suggest that removal of discarded containers in villages will allow efficient control of *Ae. aegypti,* but will likely have little impact on sylvatic arbovirus transmission because *Ae. furcifer*, the main YFV, CHIKV and DENV-2 vector in this region, primarily lays eggs in the forest and savannah.

Our data indicated that the proportion of *Ae. taylori* was much higher than that of *Ae. furcifer* in the immature fauna, while the opposite was always observed in human landing fauna [2,14,36]. This discordance suggests that a part of the population of *Ae. taylori* is not anthropophilic, or that the population of immature *Ae. furcifer* was incompletely sampled due to our failure to identify its preferred larval sites. Thus, the *Ae. taylori* population may be more important than indicated by human landing catch data. Moreover, if we consider that non-anthropophilic mosquitos are generally also non-primatophilic [37], a portion of the *Ae. taylori* population may feed in as yet unknown hosts in the forest. Our possible failure to fully identify the preferred larval sites of *Ae. furcifer* may be due to the fact that we sampled only visible and readily accessible tree holes (located at less than 2 m above the ground) while some tree holes were located more than 10 m high. These elevated tree holes may be preferred larval habitats for *Ae. taylori*; height-dependent oviposition behavior has been already observed in African forests [15,38,39] and in Indiana [40]. This hypothesis requires further investigation. Although *Ae. furcifer* was the main sylvatic arbovirus (YF, DENV-2 and CHIKV) vector collected by human landing collections and was the only species found infected in villages in Africa [2,14,41], its larvae were not found within these villages. Therefore, the adult females of this species probably invade villages each evening from savannahs and/or forests, where we found its larvae in tree holes and fruit husks. A more detailed understanding of the movement of *Ae. furcifer* between larval habitats and human habitations will yield a better understanding of how people are exposed to sylvatic YFV, DENV-2 and CHIKV.

We found *Ae. vittatus* larvae in all land cover classes and in 5 of the 9 microhabitats sampled. Its most common larval habitats were puddles followed by rock holes in this study. Although it has been already found in puddles in Midwestern Nigeria [42], the larvae of this species are generally known to occupy rock holes in Africa [43,44].

Our data agree with previous studies showing that *Ae. luteocephalus* larvae are generally found in natural tree holes and in low frequencies in various water containers in villages [24,30,39,45]. This mosquito has been collected year-round in Nigeria [39] and its larvae have been collected 4 months after the last seasonal rainfall in the Kédougou region [31]. Thus, *Ae. luteocephalus* may be considered particularly tolerant of dry conditions [39] but may stop larval development in response to a lack of wet tree holes. Our data also suggest that the *Aedes* of the africanus group found by Raymond *et al.*[13] were probably *Ae. luteocephalus*.

The preference of this species to oviposit in tree holes is in agreement with the findings of Dunn [46] and Anosike *et al.*[47] in Nigeria. However, another investigator also found this species in water containers within villages in the same country [48].

Other mosquitoes like *Er. chrysogaster* and *Ae. longipalpis* were also highly represented in the immature fauna we collected, while they were scarce or absent from all previous 25-hour [12] and crepuscular [2,14] human landing collections in the area. CHIKV or DENV-2 strains have not been isolated from these species under natural conditions, but they may be considered as potential vectors. Indeed, *Er. chrysogaster* has been shown experimentally to have a higher vector potential for CHIKV than *Ae. aegypti*[49,50]. Ae*. longipalpis* belongs to the same subgenus as *Ae. niveus* and *Ae. ingrami,* which are sylvatic DENV vectors in Malaysia [51] and potential CHIKV vectors in the Ivory coast [52], respectively. The presence of large populations of non-anthropophilic *Ae. taylori*, *Er. chrysogast*er and *Ae. longipalpis* suggest the existence of an as-yet undescribed secondary enzootic cycle of DENV-2 and CHIKV.

## Conclusions

Our study provides valuable information on the larval ecology of sylvatic arbovirus vectors in southeastern Senegal. We have shown that *Ae. furcifer* and *Ae. taylori* larvae occur mainly in tree holes in forest and savannah land covers, unlike immature *Ae. aegypti* and *Ae. vittatus,* which were found in a wider range of microhabitats and land cover classes. *Ae. luteocephalus* was collected mainly in forest and savannah land covers within tree holes and fruit husks but was also collected in a lower frequency in various containers in villages. Larvae of zoophilic *Ae. aegypti formosus* were frequently found in discarded containers in villages. We also detected a high proportion of non-anthropophilic potential vectors in the larval mosquito fauna suggesting the existence of still obscure YFV, DENV-2 and CHIKV cycles in southeastern Senegal.

Removal of discarded containers will be efficient for controlling *Ae. aegypti* in villages but will have little or no impact in *Ae. furcifer*, the principal DENV and CHIKV vector to humans.

## Competing interests

The authors declare that they have no competing interests.

## Authors’ contributions

MD, SCW, AAS and KAH conceived the study. DD designed the protocol. MB carried the remote sensing and geospatial analysis. DD and CTD carried out the field work. DD and MD analyzed the data and drafted the manuscript. DD, CTD, MB, KAH, SCW, ID, YB and MD critically revised the manuscript. All authors read and approved the final manuscript.
